# The mystery of language evolution

**DOI:** 10.3389/fpsyg.2014.00401

**Published:** 2014-05-07

**Authors:** Marc D. Hauser, Charles Yang, Robert C. Berwick, Ian Tattersall, Michael J. Ryan, Jeffrey Watumull, Noam Chomsky, Richard C. Lewontin

**Affiliations:** ^1^Risk-Eraser, LLCWest Falmouth, MA, USA; ^2^Department of Linguistics and Computer and Information Sciences, University of PennsylvaniaPhiladelphia, PA, USA; ^3^Department of Electrical Engineering and Computer Science, Massachusetts Institute of TechnologyCambridge, MA, USA; ^4^Division of Anthropology, American Museum of Natural HistoryNew York, NY, USA; ^5^Department of Integrative Biology, University of TexasAustin, TX, USA; ^6^Department of Theoretical and Applied Linguistics, Cambridge UniversityCambridge, UK; ^7^Department of Linguistics and Philosophy, Massachusetts Institute of TechnologyCambridge, MA, USA; ^8^Department of Organismic and Evolutionary Biology, Harvard UniversityCambridge, MA, USA

**Keywords:** language evolution, computation, animal behavior, genetics, paleoarcheology, modelling

## Abstract

Understanding the evolution of language requires evidence regarding origins and processes that led to change. In the last 40 years, there has been an explosion of research on this problem as well as a sense that considerable progress has been made. We argue instead that the richness of ideas is accompanied by a poverty of evidence, with essentially no explanation of how and why our linguistic computations and representations evolved. We show that, to date, (1) studies of nonhuman animals provide virtually no relevant parallels to human linguistic communication, and none to the underlying biological capacity; (2) the fossil and archaeological evidence does not inform our understanding of the computations and representations of our earliest ancestors, leaving details of origins and selective pressure unresolved; (3) our understanding of the genetics of language is so impoverished that there is little hope of connecting genes to linguistic processes any time soon; (4) all modeling attempts have made unfounded assumptions, and have provided no empirical tests, thus leaving any insights into language's origins unverifiable. Based on the current state of evidence, we submit that the most fundamental questions about the origins and evolution of our linguistic capacity remain as mysterious as ever, with considerable uncertainty about the discovery of either relevant or conclusive evidence that can adjudicate among the many open hypotheses. We conclude by presenting some suggestions about possible paths forward.

## Introduction

Inquiry into the origins of language was banned by the Société de Linguistique de Paris in 1866 because speculative flourishes far outpaced hard evidence. Within the past 40 or so years, however, writings on this subject have exploded (Lieberman, 1984; Bickerton, 1990; Pinker and Bloom, 1990; Jackendoff, 1999; Fitch, 2010; Hurford, 2011), implying that hard evidence has outpaced speculation. This perspective, shared by many, is due in part to the emergence of new techniques to study animal social behavior, decipher the fossil record, map genomes, and model evolutionary processes. The sheer abundance and public visibility of such studies, including claims of human-like cognition in birds and primates, along with talking Neanderthals, might suggest that important strides have been made in understanding the origins of human language, its precursors in other animals, the selective pressures that led to its design features and adaptive significance, as well as its genetic underpinnings. We argue instead that both scientists and journalists have rushed to premature conclusions based on woefully incomplete or absent evidence.

We begin with a brief case study to illustrate how biologists typically study the evolution of a behavioral phenotype. We then turn to a discussion of the language phenotype, including its core biological computations and representations. Next, we discuss four approaches to the evolution of language: comparative animal behavior, paleontology and archaeology, molecular biology, and mathematical modeling. In each section, we state why we consider the evidence inconclusive or irrelevant. We conclude with a brief set of empirical desiderata for moving forward, noting the limitations that lie ahead, at least for the foreseeable future.

## How to study the evolution of a trait

Understanding biological evolution requires distinguishing patterns and processes, dissecting potential contributions from both random and non-random mechanisms including genetic drift, migration, selection, developmental unfolding, and genetic constraints. Rarely do biologists have access to all of the relevant evidence, and this is especially true for higher vertebrates and the complicated social behaviors they exhibit. When it comes to human language evolution, the paucity of relevant evidence is significant and, as we discuss below, the potential for acquiring such evidence is completely closed off in some relevant areas of inquiry (e.g., no options for living sister species). This point is brought into focus by looking at far simpler systems, such as the túngara frog (Ryan, 1985; Ryan and Rand, 2003), which we turn to next.

Male túngara frogs sexually advertise with simple (whine only) or complex (whine plus chucks) mating calls. Playback experiments show that males add chucks in response to the calls of other males and that females prefer calls with chucks. Thus, males gain a reproductive benefit by making complex calls, while females gain a reproductive advantage from mating with larger males who fertilize more eggs. The reproductive gain to males is, however, partially offset by the attractiveness of chucks to frog-eating bats.

The chucks are generated by large larynges with pendulous masses extending from the vocal folds. This morphology and the chucks they produce are restricted to túngara frogs and their sister species. The tuning of the female's two inner ear organs, the AP (amphibian papilla) and BP (basilar papilla), match the dominant frequencies of the whine and chuck, respectively, and the BP tuning is better matched to and thus more stimulated by the lower-frequency chucks of larger males than their higher-pitched conspecifics (Ryan et al., 1990).

In sum, we know how these frogs communicate, the fitness costs and benefits of communication, the phylogenetic distribution of key traits, and details of the mechanisms underlying signal production, perception, and behavioral response. But how did this system evolve?

The obvious adaptive hypothesis is that females evolved their BP tuning because of the reproductive advantage of mating with larger males making lower-frequency calls. Alternatively, BP tuning represents an ancestral trait, whereby males evolved calls to exploit the female's preexisting sensory biases. Comparative evidence resolved this issue (Wilczynski et al., 2001). Only túngara frog males and their sister species add chucks to their whines. The other related species only produce whines, their whines all stimulate their AP organs, and they all have BP organs that are not recruited into the communication system. For most of these species, the tuning is statistically indistinguishable from the BP tuning of the túngara frog. These findings reject the adaptationist hypothesis in favor of the alternative: tuning existed in this clade for millions of years prior to the evolution of the chuck, and was poised to be stimulated when males eventually evolved the larynx allowing them to produce chucks. These results highlight the point that arguments based on fit and sound adaptive logic need not be correct.

In sum, evolutionary analyses demand a clear specification of the target phenotype, empirical evidence linking details of trait design features to fitness consequences, an understanding of the comparative landscape in terms of homologous and analogous traits, and tests that distinguish adaptive from non-adaptive explanations for trait diversification. This recipe for successful evolutionary analysis has rarely been followed in the case of language, and given the limited evidence available, the current prospects are not strong, especially in some domains of analysis. For example, unlike the túngara frog, there are no living sister species to test out phylogenetic hypotheses, and for both methodological and ethical reasons, no ability to manipulate particular characteristics of the language faculty to assess the impact on individual fitness. Nonetheless, the most productive way forward, we believe, is to define important details of the language phenotype, recognize generally accepted methods and evidence in evolutionary biology, and work within this framework to assess what we may learn about the evolution of language.

## The language phenotype

In this paper, we are interested in biological as opposed to cultural evolution. Given this focus, we ask: what are the core biological mechanisms that enable the capacity for language? As we and many other language scientists see it, the core competence for language is a biological capacity shared by all humans and distinguished by the central feature of discrete infinity—the capacity for unbounded composition of various linguistic objects into complex structures. These structures are generated by a recursive procedure that mediates the mapping between speech- or sign-based forms and meanings, including semantics of words and sentences and how they are situated and interpreted in discourse. This approach distinguishes the biological capacity for language from its many possible functions, such as communication or internal thought.

One approach to exploring the core biological competence for language, and thus its evolution, was set out by Hauser et al. (2002). A central focus of this paper was on conceptual and methodological issues that might help distinguish capacities that are shared with other animals as opposed to being uniquely human, as well as capacities that are uniquely human and unique to language, as opposed to shared with other domains of knowledge. These distinctions were mapped, respectively, to the Faculty of Language in the Broad (FLB) sense and the Faculty of Language in the Narrow (FLN) sense. FLB designates processes that are shared with other animals, and thus, are involved in language and other sensory-motor and conceptual-intentional processes. FLN, in contrast, describes processes that are uniquely human and unique to language. As a hypothesis, Hauser et al. proposed that FLN consists of the recursive mechanisms for discrete infinity along with mappings to the interfaces with the conceptual-intentional and sensory-motor systems. Note that this proposal is not just about the recursive operations, but about how such procedures connect to other mind-internal representations, often discussed as the systems of semantics and phonology. This is an example of an evolutionary hypothesis that focuses on the core biological competence, and lays out a proposal for empirical research. In particular, if FLN is as described, there should be no homologs or analogs in other animals and no comparable processes in other domains of human thought. To help sharpen this proposal and others, we turn next to some of the core components of the language phenotype.

Language, in all aspects, consists of abstract units of information that are organized and combined following specific computational procedures. The phoneme, originally held to be the basic unit of phonology, has been shown to further decompose into combinations of features that characterize articulatory actions, which shape the sound patterns of languages. For instance, the English past tense “ed” is sometimes pronounced as /t/ as in “kissed” but /d/ as in “hugged.” This seemingly arbitrary fact is predicted on the basis of articulatory features. If the final phoneme of the verb ends in the feature “voiced,” which involves the vibration of the vocal cords during articulation (e.g., /g/ in “hug”), the past tense “ed” is pronounced as /d/, which is also voiced. By contrast, /s/ in “kiss” is unvoiced—no vocal cord vibration—which automatically results in the similarly unvoiced /t/ for “kissed.” These phonological rules can be described as familiar IF-THEN statements in computational systems. Children unconsciously and spontaneously follow these rules that may be generalized to novel words (e.g., twerked, Googled). Importantly, all of these phonological processes are paralleled in sign language (except for those directly linked to voice articulation), highlighting the fact that the computations of language are not tied to a particular sensory modality.

Word formation also takes place by combining informational units in a stepwise process familiar in computational systems. For instance, the word “unlockable” is doubly ambiguous—meaning usable either as a functional lock or a broken lock. Such duality can be captured by differences in the logical sequence of morpheme combinations: the functional lock results from combining “un” and “lock” together to be followed by “able”—something that is possible to unlock—whereas the broken lock is derived from combining “lock” and “able” first, before “un” imposes a negative sense. Such ambiguity can be represented in a form similar to arithmetic expressions: [un-[lock-able]] vs. [[un-lock]-able], which encodes the combinatorial process in word formation.

The syntactic system of human language offers the clearest demonstration of discrete infinity, but with important constraints on the range of variation. Consider the sentence “Instinctively, eagles that eat swim.” The adverb “instinctively” describes the capacity of swimming rather than eating, even though “instinctively” is linearly closer to “eat” than “swim.” In fact, no matter how far apart “instinctively” and “swim” are from each other in terms of the number of words or linear distance, the association remains: “Instinctively, eagles that eat in the Rocky Mountains swim.” The correct semantic interpretation can be derived if we consider the logical sequence in which sentence structures are built, as in the “unlockable” example above. The relative clause “eat in the Rocky Mountains” combines with and modifies the noun phrase “eagles” first, which together as a unit combines with the verb “swim.” The resulting nested structure—a clause—is then combined with the adverb “instinctively;” or, in the arithmetic representation, [Instinctively, [eagles [that eat in the Rocky Mountains] swim]]. These results, together with those involving hundreds of other languages, reveal both that the capacity for language takes hierarchical/nested structures as the basic building blocks of syntax, and that hierarchical rather than linear distance between elements is central to syntactic computation (Berwick et al., 2011; Moro, 2013).

Lastly, the recursive mechanism is typified by three properties (see, for example, Watumull et al., 2014): computability, definition by induction, and mathematical induction. Note that recursion characterizes the mechanism, not patterns (e.g., embedding) in its outputs. Computability is reflected in a procedure that generates new and complex representations by combining and manipulating discrete symbols. The computable function must be defined by a form of induction: outputs must be carried forward and returned as inputs to generate a hierarchical structure over which can be defined complex relations (e.g., syntactic, semantic, phonological, etc.). Finally, mathematical induction is realized in the jump from finite to infinite, as in the projection from a finite set of words to an infinite set of sentences. Thus, the recursive mechanisms generate an infinite set of hierarchically structured expressions that yield interpretations at the interfaces.

The structural constraints of language, observed at all levels of linguistic representation and derived from a few familiar languages, have proven remarkably successful in the description and explanation of linguistic diversity (Chomsky, 1981; Bresnan, 2001; Roberts, 2012). From these formal systems it is possible to deduce linguistic universals as consequences, thereby generating empirical predictions. For instance, a cross-linguistic survey of 500 languages shows that every language consists of sentences based on a verb phrase surrounded by modifiers in predictable, non-varying patterns (Cinque, 1999). The aboriginal languages of Australia, once believed to be very different from other languages, can be described as a constellation of properties, each identifiable in the more familiar languages (Hale, 1992; Legate, 2001). The distribution of the structural properties of language, such as word order and agreement, do not seem to follow any cultural or historical patterns (Baker, 2001). Rather, they exhibit the same limited range of variation, a result that is consistent with a species-specific linguistic capacity.

Language ontogeny provides another important source of evidence for the biological basis of language and its particular phenotype. Language acquisition often reveals a pattern of winnowing, where a child makes use of non-target but biologically possible grammars, ultimately narrowing down to the target grammar. For instance, English-learning children may omit subjects or objects (“tickles me” instead of “he tickles me”): these forms are ungrammatical for English and do not appear in the input data, but are consistent with grammars such as Mandarin Chinese that omit discourse topics (Yang, 2002). These non-target forms are gradually eliminated, in a manner similar to the winnowing process in birdsong acquisition (Marler, 1997); see (Comparative Animal Behavior). This reveals that the child is endowed with a capacity to acquire a wide range of possible grammars, which are then selected by the linguistic data in the specific environment.

Recently, steps have been taken toward the unification of linguistic theory with the genetic, neurobiological, and cognitive underpinnings of language. These studies provide rich accounts of the computations and representations underlying the language phenotype and its acquisition, but with poor understanding of its evolution. An account of language evolution is highly deficient if it cannot account for these specific empirical results.

## Comparative animal behavior

Talking birds and signing apes rank among the most fantastic claims in the literature on language evolution, but examination of the evidence shows fundamental differences between child language acquisition and nonhuman species' use of language and language-like systems. For instance, dogs can respond to a few hundred words, but only after thousands of hours of training; children acquire words rapidly and spontaneously generalize their usage in a wide ranges of contexts (Kaminski et al., 2004; Pilley and Reid, 2011). Similarly, Nim Chimpsky, the chimpanzee that produced the only public corpus of data in all animal language studies, produced signs considerably below the expected degree of combinatorial diversity seen in two-year old children (Yang, 2013), and with no understanding of syntactic structure or semantic interpretation. Though these studies are of potential interest to understanding the acquisition of specialized, artificial skills—akin to our learning a computer language—they do not inform understanding of language evolution. Hence, we focus on two potentially more promising lines of empirical inquiry: (i) observations and experiments of naturally communicating animals and (ii) experiments assessing the computational and perceptual capacities of animals, focusing on abilities necessary for human language processing.

Researchers claim that songbirds and nonhuman primates exhibit features of communication that parallel human linguistic communication. Like human infants, some songbirds acquire their species-typical song, constrained by an innately-specified template, but requiring particular acoustic input and auditory feedback during a sensitive period of development. Songbirds and babies also progress through a babbling phase on their way to developing the adult form Doupe and Kuhl (1999), Petkov and Jarvis (2012), and both learn to string syllables together in an analogous manner prior to full articulation (Lipkind et al., 2013). These observations, generated from behavioral as well as neurobiological evidence, are interesting, but do not guide understanding of language acquisition in humans for at least two reasons: unlike human language, (i) song is a highly specialized and finite system, with the underlying neurobiology linked to one sensory channel (acoustic), and the signal itself is linked to a narrow function and hardly changes once acquired; (ii) when song syllables are combined to create longer structures, there are only limited combinatorial operations and new creations have no impact on the function or “meaning” of the song. Students of child language acquisition thus rarely turn to work on songbirds for insights, except to make the very general point that there are analogous learning processes in early development.

Research on nonhuman primates has focused more on how sounds are produced than how they are acquired because our closest relatives exhibit no parallels (genetically, neurobiologically, and behaviorally) with child language acquisition: there is no vocal learning, no babbling, no sensitive period, no inductive leaps. Nonhuman primates do, however, have a vocal apparatus that is closer to our own species than that of songbirds. Nonhuman primates produce rhythmic articulations, generating sounds with formant structures (resonances that reflect the filtering properties of the vocal tract), to which they are perceptually sensitive (Fitch, 2006; Yip, 2006). Together, these observations have led to the conclusion that we share with other primates several key mechanisms for vocal articulation and sound perception, and thus, that the origins of human speech production can be traced to ancestral primates. But there is no evidence that monkeys and apes configure their repertoire on the basis of distinctive features that map to distinct articulatory gestures that, in turn, generate acoustic or visual signals. Further, both neurobiological and behavioral studies show that nonhuman primates have extremely poor voluntary control over the structure of their vocalizations, as shown by studies that have failed to operantly condition monkeys to alter spectral properties of their species-specific calls (Hauser, 1996; Jurgens, 2002; Fitch, 2010). These points are critical to understanding the nature of phonology and its externalization in speech or sign (Yip, 2006).

Other studies have explored the possibility that nonhuman animals produce vocalizations or gestures that are like our words—that is, symbolic or referential—and with the capacity for combination based on some syntactic principles (von Frisch, 1967; Seyfarth et al., 1980; Gould and Towne, 1987; Zuberbuhler et al., 1999; Manser, 2013). The classic work in this area focused on honeybee dances and vervet monkey alarm calls, with other taxonomic groups added over time. The general evidence is that when animals confront particular situations, say a predator, a dominant attacker, or the discovery of highly coveted food, they produce distinctive vocalizations or visual gestures while others respond as if the triggering context were present, causing flight, submission, or movement to food.

The question of interest is whether these seemingly modest claims about animal signals help us understand the evolution of our capacity to represent words, including not only their referentiality but their abstractness, their composition via phonology and morphology, and their syntactic roles. Our simple answer is No, for five specific reasons: for animals, (i) acquisition of the entire lexicon is complete by the end of the early juvenile period, and for most species, the sounds or gestures are innately specified; (ii) those sounds and gestures refer, at best, to directly observable objects or events, with great uncertainty about the precise meaning, and no evidence for signals that map to abstract concepts that are detached from sensory experiences; (iii) with a few rare exceptions, individuals only produce single utterances or gestures, never combining signals to create new meaning based on new structures; (iv) utterances are holistic, with no evidence of complex syntactic composition derived from an inventory of discrete morphological elements; (v) the utterances or gestures are not marked by anything remotely resembling grammatical classes, agreement, etc. Given these differences, it is not possible to empirically support a continuity thesis whereby a nonhuman animal form served as a precursor to the modern human form.

The second approach to exploring possible evolutionary precursors to language focuses on the capacity to process patterned sequences that map to different generative rules that capture some elements of human syntax. The first experiment (Fitch and Hauser, 2004) along these lines was based on the Chomsky (1959) hierarchy of formal grammars, a perspective that reveals how different procedures can generate different levels of structural complexity. This experiment tested cotton-top tamarin monkeys, comparing the two lowest levels of the Chomsky hierarchy, each necessary but insufficient to explain the full richness of linguistic competence. Using a habituation-discrimination method that is common in studies of child language acquisition, results showed that monkeys spontaneously (no training) discriminated the lowest level grammar, but not the next level up. Where they failed suggested that monkeys (at least tamarins) may not be able to spontaneously compute grammars that generate embedded patterns of output, a feature of human syntactic competence. Although embedding is certainly part of our linguistic competence, it is a computation that is far too limited to explain the richness of our syntactic capacity. Consequently, the authors concluded, some of our closest living relatives are far too impoverished, computationally, to provide insights into our own evolutionary history.

Subsequent studies (Gentner et al., 2006; van Heijningen et al., 2009; Abe and Watanabe, 2011; Rey et al., 2012) focused on the problem of embedding, virtually all used methods of extensive training, and all mistakenly equated embedding with both recursion and the claim that any evidence of embedding would rule out earlier claims of human uniqueness. For example, in one study of starlings and one on baboons, subjects were trained for months in tens of thousands of reinforced trials to learn a pattern of embedding that was comparable to that tested on tamarins. Both species learned this pattern, with limited generalization to novel patterns. The researchers concluded that recursive computations are not unique to humans, and so our competence can be explained by non-linguistic processes. For at least four reasons, however, these results do not inform our understanding of human language competence: (i) recursion, as realized in the language faculty, is a set of properties defining the generative procedure, not its output, and so should not be equated with embedding (see section The Language Phenotype); (ii) human language acquisition does not involve training with reinforcement; thus, even if the results showed parallel competences, the acquisition process and underlying computations would be entirely different; (iii) even if animals can process embedded structures, the generalization results show that the capacity is limited to the training level, and pales relative to human competence, especially if one removes some of the working memory constraints; (iv) as the Chomsky hierarchy perspective reveals, embedding is far too weak to explain human language competence, and thus, even strong evidence in animals would contribute little to our understanding of human language evolution.

What would be interesting—as a step toward addressing the definition of recursion sketched in section The Language Phenotype—would be to develop a robust and spontaneous method showing that animals can extract a generative procedure that underpins a pattern of structured inputs, and use this procedure to generalize far beyond the input. We return to this possibility in our final section.

For now, the evidence from comparative animal behavior provides little insight into how our language phenotype evolved. The gap between us and them is simply too great to provide any understanding of evolutionary precursors or the evolutionary processes (e.g., selection) that led to change over time.

## Paleontology and archaeology

Given the phenotypic characterization of language (II), it is no surprise that direct prehistoric traces of language, spoken or signed, are lacking. Consequently, those interested in language origins have often tried to document putative proxies for language in the fossil and archaeological records (Tattersall, 2012; Dediu and Levinson, 2013; Johansson, 2013). Such proxies have been of two kinds: anatomical (including genomic) and behavioral. Since neither the generation of language nor its lack leaves any identifiable imprint on brain endocasts, proxies of the first sort are limited to the preserved bony structures associated with the production and reception of articulate speech, and to alleles purportedly associated with language. The gestural origins theory of language (Corballis, 2003; Studdert-Kennedy and Goldstein, 2003), in which signed expressions predated spoken ones, is on even less stable ground: though the use of the hand in this context was possible far back in hominid evolution, the hand and its motor correlates did not evolve for this purpose, and there are no relevant comparative or fossil findings to illuminate mental representations and computations.

Due to its relative recency and the completeness of its fossil and genomic evidence, we know more about *Homo neanderthalensis* than most extinct hominids; and because of their evolutionary proximity to humans, Neanderthals provide one of the most intriguing test cases for exploring the antiquity of the language phenotype. The ability of *H. neanderthalensis* and other extinct hominids to produce the sounds that *Homo sapiens* uses in speech today has both been denied from the bony structure of the roof of the upper vocal tract, and affirmed from the occasionally preserved anatomy of the hard portion of the hyoid apparatus (Laitman et al., 1979; Lieberman, 1984; Arensburg et al., 1989; Martinez et al., 2004). Other suggestions include that Neanderthals probably spoke because they had the aural ability to process the sound frequencies associated with speech, and that they might have had language because their genome included the modern human variant of the FOXP2 gene (see Molecular Biology), known to play a role in speech articulation, among other things (Martinez et al., 2004; Krause et al., 2007; Dediu and Levinson, 2013). However, while the modern versions of each of these attributes may be necessary for speech production and comprehension, none can be regarded as a sufficient condition for inferring speech, let alone language (Tattersall, 2012).

Two other observations highlight the limitations of this research. First, acoustic perception is a highly conserved trait within primates, such that chimpanzee hearing is basically identical to ours. In contrast, vocal tract anatomy has changed significantly. Consequently, though other primates can hear what we hear, they can't produce many of our essential articulatory gestures. This demonstrates that perception and production did not coevolve, leaving claims about Neanderthal capacity completely uncertain. Second, recent studies suggest that approximately equal proportions of the horizontal and vertical sectors of the vocal tract are necessary for speech production (Lieberman, 2011). This conformation is present in *Homo sapiens* alone, as a result of the autapomorphic retraction of its face below the neurocranium. This points to a critical change after divergence from the Neanderthals. Additionally, Neanderthals (and Denisovans) appear to have lacked other alleles (CNTAP2, ASPM, MCPH1, PCDH11YandX) allegedly associated with language (see section Molecular Biology), pointing to significant molecular changes, and presumably, different selective pressures. The fossil evidence thus stands mute on the issue of central language capacity, and ambiguous at best on the question of its externalization in speech.

Another line of paleontological evidence comes from cranial endocast size and shape. Approximately 2 million years ago, following the emergence of the genus *Homo*, cranial capacity began to expand. Eventually, Neanderthal brain size was larger than ours today. Many have interpreted this consistent expansion as indicative not only of substantive changes in cognitive ability, but of the capacity for language. In particular, given the comparable cranial capacities of *Homo neanderthalensis* and *H. sapiens*, many have concluded that both had language. As such, the antiquity of the language phenotype can be traced back at least as far as the Neanderthals (Dediu and Levinson, 2013; Johansson, 2013). But as Lenneberg (1967) long ago noted, and as many more recent neurological and genetic studies have affirmed, raw brain size provides little to no insights into the computations and representations of language, either in terms of deficiencies or advantages (Price et al., 2010; Schoeneman, 2012). For example, autistics have significant problems in both the acquisition and expression of language, and yet early in development often have larger brains than healthy children. Similarly, children with one hemisphere removed prior to the full acquisition of language often display normal language expression and comprehension. These findings emphasize that a large brain is no predictor at all of language capacity or competence.

Turning to archaeology, the relevant record starts at about 2.5 million years ago—well after the origin of the hominid family—with the deliberate production of stone tools (Semaw et al., 1997). These do not in themselves tell us anything about syntax, semantics, phonology, or their interfaces, as it is abundantly clear that the manufacture of even quite complex stone tools is not necessarily associated with modern cognition. The same applies to other cognitively complex expressions such as the controlled use of fire, the manufacture of compound tools, and even the simple burial of the dead. What does appear to be significant for early cognitive style, however, is the pattern of innovation. Following the initial invention of stone tools, refinements in technology emerged sporadically. There is no hint until very recently of the pattern of continual enhancement typical of modern linguistic *Homo sapiens*. Although technologies became more complex over the history of the genus *Homo* (Tattersall, 2012), indications of modern-style iconic and representational activities (Henshilwood et al., 2002, 2004) begin only significantly after the first anatomically recognizable *H. sapiens* appears at a little under 200 thousand years ago (White et al., 2003; McDougall et al., 2005). Indeed, the sketchy archaeological traces of the earliest *Homo sapiens* in Africa are remarkably archaic. Further, despite recurrent claims to the contrary, there is no firm evidence for “modern” behaviors on the part of *Homo neanderthalensis* or any other extinct hominid species (Klein, 2009; Bar-Yosef and Bordes, 2010; Higham et al., 2010).

Whether or not language is principally an instrument for thought, we have no substantive reason to suspect language use by the hominids that preceded us. In striking contrast to the Cro-Magnon *Homo sapiens* populations that replaced it in Europe, even the highly encephalized *Homo neanderthalensis* failed to leave any unequivocal evidence for the symbolic behavior patterns (including painted and engraved imagery, the use of musical instruments and symbolic and notational systems) that characterize modern, linguistic, human beings. Neanderthal material productions represented at best an incremental increase in complexity relative to those of their predecessors. The artifactual record of contemporaneous Middle Stone Age sapiens in Africa after about 100k year ago tells a very different story, a qualitative transformation in behavior that was reflected in the earliest symbolic objects, complex planning, multi-stage technologies, and other anticipations of Cro-Magnon cognitive prowess.

As we know from the acquisition of language by small-brained babies and even individuals with pathologically small brains (Lenneberg, 1967), language is clearly independent of crude brain mass. It is presumably the product of a complex and specific internal wiring, and not simply some slowly-evolved gross by-product of increasing encephalization. Indeed, greater encephalization characterizes several independent lineages within the genus *Homo*, without substantive archaeological indications of symbolic and putatively linguistic behaviors except in our own case.

In summary, the paleontological evidence is silent with respect to the capacity for both the internal computations and representations of language and its externalization in linguistic expression and communication. As we note in our final section, it is conceivable that methodological advances will enable a more fine-grained understanding of internal neurobiological structure from details of skull structure, but we are nowhere near such discoveries at present. Archaeological evidence, in contrast, points to the emergence of a language of thought in early *Homo sapiens*, replete with symbolic representations that were externalized in iconic form. We know nothing, however, about when the relevant syntactic and semantic machinery evolved, what selective pressures—if any—were responsible for its emergence, and when such internal computations were externalized in spoken or signed language. Whenever this occurred, present evidence suggests it was after our divergence with Neanderthals, and thus, a very recent event.

## Molecular biology

The comparative method provides an important approach to identifying genetic mechanisms and evolutionary change, but runs into significant challenges in the case of language. To illustrate, consider FOXP2, an autosomal dominant transcription factor that has been linked to the Mendelian disorder verbal dyspraxia SPCH1, and so a gene apparently necessary, but crucially not sufficient, for human speech, let alone language. The human variant of this gene differs from chimpanzees and gorillas in just two amino acid coding positions (Enard et al., 2002), while humans and Neanderthals do not differ at all. However, as noted in our section Paleontology and Archeology, it is not possible to draw firm inferences from the Neanderthal genome since we lack evidence for the relevant behavioral signals. Moreover, it is now unclear whether the putative adaptive evolutionary changes in FOXP2—perhaps related to speech—were in fact centered on these two protein coding regions as opposed to non-coding regions of FOXP2 which are arguably different between humans and Neanderthals (Maricic et al., 2012). Comparative work with birds and mice has provided some insight regarding the functioning of the FOXP2 transcription factor in zebra finch vocal learning (Haesler et al., 2007; Scharff and Petri, 2011) as well sound production in mice (Enard et al., 2009), and more recently, neural growth in mice (Tsui et al., 2013). However, these results are still far removed from the computations and representations that underlie the language phenotype.

As predicted by King and Wilson (1975), the fact that there are remarkably few protein differences between humans and chimpanzees implies that the uniquely distinguishing differences between our species might be better attributed to regulatory changes (both cis and trans) along with other non-protein coding differences [e.g., non-coding RNA, microRNAs, methylation patterning, chromatin epigenetic effects (Carroll, 2005; Somel et al., 2013)]. The results to date regarding specific human-language related genes have generally confirmed this expectation, as emphasized in a recent, comprehensive review by Geschwind and Rakic (2013 p. 638) noting that there are “only about two dozen genes estimated to be present in human… and not in chimpanzee.”

To be sure, steady progress is being made in building a more complete “parts list” of the genome-implicated changes in the hominid lineage that ultimately resulted in the substrate for human cognitive abilities, including regulatory genomic changes. The catalog of differences has grown to include the possible genomic underpinnings for neoteny (MEF2A, Somel et al., 2013); distinct brain architecture and development, such as novel cortical layer architecture and gyri (Bae et al., 2014); novel neuronal cell types, evolutionary duplication of developmental proteins (SRGAP2) resulting in novel dendritic spine density and form (Dennis et al., 2012; Geschwind and Rakic, 2013), and so forth. Thus the possible avenues for explanation are expanding, an indication of positive paths forward (e.g., see Boeckx and Benítez-Burraco, 2014). Nonetheless, even in the best understood cases, the genotype-phenotype gap remains large. Constructing bridge theories becomes more difficult because we must unravel regulatory networks not directly “wired” to phenotypes. Konopka et al. (2012) have begun unraveling this structure by a functional, modular analysis of the human vs. nonhuman primate transcriptome, but much remains unknown. Further, language is not like the examples of body plan segmentation or eye formation where functional and developmental processes are well understood in numerous species, both closely and distantly related. There are simply no precise analogs or homologs of human language in other species.

Given current limitations, genomic-driven analysis of language has resorted to roughly four research strategies: (i) locating candidate genes based on the genomic signatures of rapid, recent evolutionary change or uniqueness in the *Homo* lineage as compared to the closest nonhuman primates; (ii) exploiting language-related aberrant phenotypes, generally at the “input-output” interfaces of human language (e.g., speech and hearing), proceeding as in the genomic analysis of complex human pathologies; (iii) using twin studies and/or SNP analysis to search for genes correlated with normal human variation; (iv) developing nonhuman animal models, as in the work with mice, while setting aside the absence of a full nonhuman language phenotype to examine plausible subcomponents such as transcriptional neural plasticity and motor control.

The FOXP2 case exemplifies research strategies (i), (ii), and (iv). FOXP2 is a transcription factor that up- or down-regulates DNA in many different tissue types (brain, lung, gut lining) at different times during development as well as throughout life. This broad functional effect makes evolutionary analysis difficult. In particular, the exact mechanisms by which FOXP2 mutations disrupt speech remain uncertain, variously posited as disruptions in motor articulation/serialization in speech, vocal learning generally, or broader difficulties with procedural serialization. This is critical because FOXP2 mutations may disrupt only the input/output systems of language, sparing the more internal computations of human language syntax or semantics; or it may be that FOXP2 affects general cognitive processing, such as general serial ordering of procedures. Second, it is not clear whether the amino acid changes distinguishing FOXP2 in humans and nonhumans represent adaptations “for” language, since their functional effects remain unclear. One of the two protein-coding changes along the lineage to modern humans is also associated with the order Carnivora. Since FOXP2 also targets the gut lining, this evolutionary step may have had little to do directly with language but instead with digestion modifications driven by forest-to-savannah habit and so dietary change (Zhang et al., 2002), as in the well-established case of lactose tolerance and dairy culture (Bersaglieri et al., 2004).

FOXP2's many downstream regulatory targets have also been associated with other language disorders; for example, CNTNAP2, a neurexin-family neural growth factor, appears linked to autism and specific language impairment (SLI) (Vernes and Fisher, 2011; Vernes et al., 2011). Note that CNTNAP2 itself is also regulatory, in line with the King and Wilson prediction, but unlike FOXP2, is different in humans and Neanderthals. Moreover, its link to SLI is not clear-cut. Rice (2012) presents evidence that SLI might best be pictured as a disruption of a growth timing mechanism, implicating a different set of genetic components, some that are non-regulatory and involved in neuronal migration. The story is not yet complete; as Geschwind and Rakic (2013) note, comparison of human vs. mouse FOXP2 by Tsui et al. (2013) points to a role beyond neural circuit construction, to neural cell proliferation itself.

Together, these observations underscore the fact that we lack a connect-the-dots account of any gene to language phenotype. Furthermore, to the extent that our account of the language phenotype is diffuse (some general system of cognition rather than a precisely delimited and narrow computational module), the genotype-phenotype mapping will be correspondingly more challenging to address. Given our currently impoverished understanding of such mappings for far less complicated phenotypes, in far simpler organisms, molecular biology has a long way to go before it can illuminate the evolution of language.

## Modeling

Biological models of language evolution often start with a population of individuals communicating by means of their particular languages, broadly defined as mappings between forms and meanings. A certain measure of fitness is introduced, which in turn differentially affects the transmission of the languages to the next generation of individuals. This evolutionary dynamic is believed to shed light on the emergence of human language and its associated properties. A notable result (Nowak et al., 2001), congruent with the comparative linguistics and mathematical learning theory, suggests that if the reliability and efficiency of language learning is the fitness metric, the space of possible languages must be limited in its size and complexity for language to emerge.

The vast majority of modeling efforts, like those above, presuppose the existence of a language phenotype equipped with compositionality and discrete infinity. This assumption is directly built into the mathematical models (Nowak and Komarova, 2001; Kirby and Hurford, 2002) or enabled by human subjects in behavioral studies, who may impose linguistic structures upon the materials presented (Kirby et al., 2008). But this presupposition regarding the language phenotype offers no insight into how it arose in the first place, nor does it illuminate the fundamental distinction between the emergence of the core biological competence and its adaptive or non-adaptive functions. Lastly, the underlying assumptions of these models, including their commitment to an adaptationist program, are often made without empirical verification and in some cases, are contrary to known facts about languages. As noted in our section How to Study the Evolution of a Trait, it is essential for proposals of adaptive function to be tested against non-adaptive hypotheses.

A leading proposal in evolutionary modeling is to identify language fitness with communicative success (Nowak and Komarova, 2001; Baronchelli et al., 2012): individuals who communicate with and learn languages more successfully have greater reproductive success. While our pre-linguistic past is not accessible for direct investigation, the uniformitarian principle of historical science does enable us to test these assumptions: If communication has played a significant role in the evolution of language, its force should be observable in the process of language transmission. The history of language change provides the only testable case for the predictions of this communication optimization thesis, and the evidence points in the opposite direction.

Language change generally proceeds mechanically irrespective of communicative purpose, a perspective held by traditional historical linguistics and strengthened by the quantitative study of ongoing language variation and change (Labov, 1994). For instance, one of the most robustly attested linguistic changes is phonemic merger, whereby the distinction between two consonants or vowels is lost. In many dialectal regions of North American English, the vowels in “cot” and “caught” are the same, while the distinction is retained in other regions. Mergers, by definition, obliterate the distinction between words, which increases the ambiguity of communication; unsurprisingly, therefore, information theoretic accounts of phonemic change have been unsuccessful (King, 1967; Surendran and Niyogi, 2006). Yet mergers can spread rapidly across dialect boundaries and are rarely, if ever, reversed (Labov, 1994). Another major difficulty with the communication as adaptation thesis can be observed in the redundancy and reduction of linguistic information. For example, the word final consonants /t/ and /d/ in English are sometimes omitted in speech so “walked” is pronounced as “walk.” In a sentence such as “I have walked home”, the perfective meaning is doubly expressed by the auxiliary “have” and “by” the verb. In the simple past “I walked home”, only the final “d” on the verb conveys the temporal information. A communication-based approach to language use would predict a higher rate of deletion for the past participle than for past tense on grounds of communicative efficiency, yet the deletion rates do not differ in these contexts (Guy, 1991).

Under the adaptationist assumption in language evolution modeling, languages that facilitate more efficient communication are more successful in transmission to the next generation. But there is no evidence of a communicative advantage for typologically more common, and thus more successfully transmitted languages such as those with the Subject Verb Object order (e.g., English) over those with the rare word order of Verb Object Subject (e.g., Malagasy). Likewise, the cross-linguistic studies of language acquisition show a largely uniform developmental trajectory, with no evidence to suggest that some languages are easier to learn than others (Slobin, 1985). While the differences between individuals' language learning abilities may have a genetic basis, there is currently no evidence to support a higher biological fitness for the more proficient learners, except in extreme cases of neurological impairment. That communication is intimately related to language is too obvious to dismiss entirely, but its lack of theoretical constraint and repeated failures, long recognized in the empirical study of language, cast serious doubt on its utility in models of language evolution.

The success of evolutionary population genetics lies in the mutually constraining connection between idealized models and empirically grounded work in the laboratory and in the wild. As noted in section How to Study the Evolution of a Trait, many tools and methodologies available to biological investigations cannot be applied to the study of language. But conceptual confusion and detachment from the empirical research of language, as we have seen in the modeling work on language evolution, unsurprisingly provides scant insights into language origins or its subsequent evolution. Moving forward, modeling work must focus on the computations and representations of the core competence for language, recognize the distinction between these internal processes and their potential externalization in communication, and lay out models that can be empirically tested in our own and other species. This is a tall order, but a necessary one if the fruits of evolutionary modeling that have been reaped from studies of, for example, mating behavior and cooperation, can be obtained for the language phenotype.

## Conclusion

Answering evolutionary questions is of profound interest largely because of our deep-seated curiosity about the past, about how things were, and how they have become what they are. Thanks in part to the revolution that Darwin sparked, including his ideas and methods, we now have many fine examples in which theoretical predictions about the origins and subsequent evolution of a phenotype have been described in great detail, including analyses of genomes, anatomy, and behavior. And yet some phenotypes remain poorly understood, and may remain so due to inadequate methods and impoverished evidence.

The evolution of our language phenotype may remain stubbornly resistant to empirical inquiry, and yet, as indicated in Table 1, there are potential empirical prospects, some near term, others quite remote. We conclude with a brief discussion of potential paths forward.

**Table 1:** **Some prospects for future empirical work on language evolution**.

**Topic**	**Description**
**COMPARATIVE ANIMAL BEHAVIOR**	
Natural communication	• One of the most empirically grounded areas comes form the study of acoustic and gestural communication, as these are tied to relatively objective empirical evidence. Though even primate articulation is, in many respects, different from human articulation, there are prospects for defining a form of phonology, both visual and acoustic, in terms that are familiar to phoneticians, and then modeling how this system may have changed based on inferences of changes in vocal tract morphology and control over hand articulation.
	• To date, our understanding of communicative signals, what they might mean or refer to, is largely limited to field studies that map sound to context and then use playbacks to explore crude behaviors; such methods would fail to enlighten human inquiry. New methods must therefore be explored, especially ones that can tap into captive settings where details of the stimuli and environment are readily controlled. Since many of the species that have been explored in this context (e.g., chimpanzees, monkeys, dolphins) can be studied in captivity, this is a potential growth area.
Artificial language	• One of the biggest drawbacks to work in this area is methodological, as most of the studies employ operant conditioning with massive training, followed by what is typically weak generalization. Alternative methods, such as habituation-discrimination, have the virtue of spontaneity, but are limited by the need for large sample sizes, and typically, new test subjects for each test. If alternative methods can be found, such as the possibility of neural recordings in free-moving animals, sampling something akin to mismatch negativity, progress is likely. Such progress will, however, require evidence of spontaneous generalization far beyond the input, varying both the nature of the stimuli, moving toward something approximating discrete infinity.
**PALEONTOLOGY AND ARCHAEOLOGY**	
Skulls and bones	• To date, little can be inferred from skull size; if we found a modern human skull, we would not know if this individual was normally functioning or suffered from some form of brain damage or developmental disorder. However, there may come a day when subtle aspects of skull structure inform some aspect of brain function, and perhaps this might illuminate changes in capacity; we say this with caution, however, given that current work in neuroimaging reveals intricate circuitry, far below the surface structures of the brain. Similarly, perhaps more detailed study of the structure of the hyoid bone, together with jaw morphology, will speak to the possible phonological spaces that are possible, and this will inform potential changes in speech production.
Artifacts	• Much of our understanding of the archaeology is restricted to a fairly recent time period, making evolutionary analyses limited. However, archaeologists continue to unearth novel evidence, including especially from the Neanderthals. This leaves open the possibility of finding more ancient evidence of symbolic activity, perhaps less iconic forms, and perhaps with ordered structures.
**MOLECULAR BIOLOGY**	
Gene-behavior mapping	• The biggest problem in this area is not specific to the problem of language, but rather to our ability to map genes to complex behavior. This state of affairs is bound to improve, especially given the rapid progress in deriving complete genomes of even extinct species, and of creating transgenics. This will open the door, especially with model organisms, of knocking in or out genes from other species with known capacities; though the ethical issues for nonhuman primates will remain, it is tempting to think about what would happen if genes known to impact human language function could be inserted into a developing ape brain.
Selection	• As increasing knowledge of the primate genomes grows, together with *H. neanderthalensis* and *H. sapiens*, we will be in a stronger position to explore the role of mutations and selective sweeps that may have contributed to change; this of course depends on deeper knowledge of the mappings between genes and relevant linguistic processes.
**MODELING**	
Focus on computations and representations	• There is a need to model how the core processes of generative computation, together with syntactic, semantic, and phonological representation, arose, and what may have selected for them. These need to be realistic models, or minimally, models that can be tested with respect to the plausibility of their assumptions.

Animal communication systems have thus far failed to demonstrate anything remotely like our systems of phonology, semantics, and syntax, and the capacity to process even artificially created stimuli is highly limited, often requiring Herculean training efforts. Should new methods reveal more richly structured systems of communication or more powerful, spontaneous abilities to process strongly generated stimuli, then comparative data would gain greater interest and relevance to evolutionary understanding. For example, we can imagine that in the not so distant future, it will be possible to non-invasively obtain neural recordings from free-ranging animals, and thus, to provide a more fine grained and quantitative measure of spontaneous processing of different stimuli. This would solve the methodological desiderata of creating a technique that reveals a capacity in the absence of reinforced training. With this tool, future work on artificial language processing might develop a set of stimuli that are generated from a recursive operation such as Merge (a recursive operation that combines two objects, such as two lexical items, to construct a new object, such as a phrase, in a process that can be iterated indefinitely), expose animals to a subset of these, and then test them on a wide range of alternatives that extend beyond the initial set in ways that can reveal substantial generalization, and thus comprehension of the underlying generative operation. As in all such studies, it would be necessary to show that simpler, finite mechanisms, cannot account for the patterns of generalization.

With respect to paleontology, it is difficult to imagine how any kind of fossil evidence could shed light on the computations and representations of language: as noted, peripheral anatomy without soft tissue says little about either the output or the phonological representations, and endocasts say even less about potential computations and representations. Nonetheless, it is not inconceivable that finer-grained analyses of endocasts from modern humans might be linked to more fine-grained neurobiological structures at the surface, and that these in turn might reveal details of the internal circuitry. That said, it is important to note here that our current understanding of how neurobiological systems link to even “language-like” communication in animals is, at best primitive, and is absent when it comes to the core competences of language in humans. For example, despite the relative simplicity of the honeybee's brain, we know nothing about how neurons encode the perception of the waggle dance, or how neurons generate motor sequences for dancing. For our own species, we know nothing about the neurobiology of our recursive procedures, and even for such seemingly simpler systems, such as phonology, our understanding is very poor (Poeppel, 2012). Needless to say, this makes comparative work virtually impossible as the target circuitry for modern humans is unclear. As advances in neuroimaging and other cellular techniques improve, so too perhaps will understanding.

In terms of the archaeological record, we can certainly imagine the discovery of richer symbolic artifacts, perhaps even non-iconic strings of symbols, dating before the emergence of *Homo sapiens*. Such findings would push back the origins of symbolic capacities, and provide greater traction into questions of both origin and subsequent evolution.

Should such discoveries from comparative animal behavior, paleontology, neurobiology, and archaeology be made, along with greater depth of understanding of gene-phenotype mapping, it would open the door to more relevant genomics and modeling. These are all big IFs about the nature and possibility of future evidence. Until such evidence is brought forward, understanding of language evolution will remain one of the great mysteries of our species.

## Author contributions

All authors contributed to the writing of this review.

### Conflict of interest statement

The authors declare that the research was conducted in the absence of any commercial or financial relationships that could be construed as a potential conflict of interest.
